# Relationship between body adiposity measures and risk of primary knee and hip replacement for osteoarthritis: a prospective cohort study

**DOI:** 10.1186/ar2636

**Published:** 2009-03-05

**Authors:** Yuanyuan Wang, Julie Anne Simpson, Anita E Wluka, Andrew J Teichtahl, Dallas R English, Graham G Giles, Stephen Graves, Flavia M Cicuttini

**Affiliations:** 1 Department of Epidemiology and Preventive Medicine, Monash University, Central and Eastern Clinical School, Alfred Hospital, Melbourne, VIC 3004, Australia; 2 Centre for Molecular, Environmental, Genetic and Analytic Epidemiology, School of Population Health, University of Melbourne, Carlton, VIC 3053, Australia; 3 Cancer Epidemiology Centre, The Cancer Council Victoria, Carlton, VIC 3053, Australia; 4 Baker Heart Research Institute, Commercial Road, Melbourne, VIC 3004, Australia; 5 Department of Orthopaedic Surgery, University of Melbourne, Royal Melbourne Hospital, Parkville, VIC 3050, Australia; 6 AOA National Joint Replacement Registry, Discipline of Public Health, School of Population Health & Clinical Practice, University of Adelaide, SA 5005, Australia

## Abstract

**Introduction:**

Total joint replacement is considered a surrogate measure for symptomatic end-stage osteoarthritis. It is unknown whether the adipose mass and the distribution of adipose mass are associated with the risk of primary knee and hip replacement for osteoarthritis. The aim of the present investigation was to examine this in a cohort study.

**Methods:**

A total of 39,023 healthy volunteers from Melbourne, Australia were recruited for a prospective cohort study during 1990 to 1994. Their body mass index, waist circumference, and waist-to-hip ratio were obtained from direct anthropometric measurements. The fat mass and percentage fat were estimated from bioelectrical impedance analysis. Primary knee and hip replacements for osteoarthritis between 1 January 2001 and 31 December 2005 were determined by data linkage to the Australian Orthopaedic Association National Joint Replacement Registry. Cox proportional hazards regression models were used to estimate the hazard ratios (HRs) for primary joint replacement associated with each adiposity measure.

**Results:**

Comparing the fourth quartile with the first, there was a threefold to fourfold increased risk of primary joint replacement associated with body weight (HR = 3.44, 95% confidence interval (CI) = 2.83 to 4.18), body mass index (HR = 3.44, 95% CI = 2.80 to 4.22), fat mass (HR = 3.51, 95% CI = 2.87 to 4.30), and percentage fat (HR = 2.99, 95% CI = 2.46 to 3.63). The waist circumference (HR = 2.77, 95% CI = 2.26 to 3.39) and waist-to-hip ratio (HR = 1.46, 95% CI = 1.21 to 1.76) were less strongly associated with the risk. Except for the waist-to-hip ratio, which was not significantly associated with hip replacement risk, all adiposity measures were associated with the risk of both knee and hip joint replacement, and were significantly stronger risk factors for knee.

**Conclusions:**

Risk of primary knee and hip joint replacement for osteoarthritis relates to both adipose mass and central adiposity. This relationship suggests both biomechanical and metabolic mechanisms associated with adiposity contribute to the risk of joint replacement, with stronger evidence at the knee rather than the hip.

## Introduction

Total joint replacement is a very effective treatment for end-stage, symptomatic knee and hip osteoarthritis (OA), and has been considered a surrogate measure for severe OA. The rate of joint replacement is steadily increasing in most developed countries [1,2]. Major risk factors for OA are age and obesity [3]. In developed countries, the populations are steadily aging and obesity rates are increasing. Chronic diseases related to aging and obesity, such as OA, will therefore probably become more prevalent and will in turn increase the demand for definitive therapies, such as joint replacement surgery.

Obesity has been recognized as the most important modifiable risk factor for OA. Although there is strong evidence to suggest that obesity is associated with the risk of knee OA and that weight loss reduces the risk of developing knee OA [4-6], the evidence for the relationship between obesity and risk of hip OA has been less consistent [7-12]. The body mass index (BMI) is the most commonly used measure of obesity, and there is accumulating evidence demonstrating a positive association between BMI and the risk of both knee [13,14] and hip [13-17] replacement. The BMI does not account for the pattern of fat distribution or body composition, however, and cannot discriminate adipose from nonadipose body mass [18]. Moreover, although the waist circumference and the waist-to-hip ratio (WHR) estimate central adiposity, and have been shown to be better predictors of several chronic diseases such as diabetes and cardiovascular diseases than the BMI [19], the role of central adiposity in the risk of joint replacement has not been fully elucidated.

A recent population-based prospective cohort study demonstrated increased incidences of knee and hip joint replacement due to OA in relation to different body mass measures, including BMI, waist circumference, WHR, weight, and percentage of body fat. The BMI was associated with much higher relative risk than were the WHR or percentage fat, suggesting a major link between overweight and biomechanics in increasing knee and hip OA risk, but did not exclude a contributing role of metabolic factors associated with adipose tissue [20]. Whether the adipose mass and the distribution of adipose mass are associated with the risk of knee and hip joint replacement secondary to severe OA, however, has not been fully elucidated.

In the present study we examined the relationship between different adiposity measures and the risk of subsequent primary knee and hip joint replacement for OA in a prospective cohort study over 10 years.

## Materials and methods

### The cohort

The Melbourne Collaborative Cohort Study (MCCS) is a prospective cohort study of 41,528 people (17,049 men) aged between 27 and 75 years at baseline, 99.3% of whom were aged 40 to 69 years [21]. Participants were recruited between 1990 and 1994 via Electoral Rolls (registration to vote is compulsory for Australian adults), advertisements, and community announcements in the local media (for example, television, radio, newspapers). Southern European migrants to Australia (including 5,425 from Italy and 4,535 from Greece) were deliberately oversampled to extend the range of lifestyle exposures and to increase genetic variation. The study protocol was approved by The Cancer Council Victoria's Human Research Ethics Committee.

Follow-up was conducted by record linkage to Electoral Rolls, electronic telephone books and the Victorian Cancer Registry and death records. From 2003 onwards, 28,046 study participants (68% of the original MCCS participants) took part in the second follow-up.

### Study participants

Of the 41,528 participants recruited, 2,505 (6.0%) were excluded from analysis because they: died or left Australia prior to 1 January 2001; had undergone a sex change since baseline; reported at the MCCS second follow-up a primary joint replacement prior to 1 January 2001; left Australia before the date of having a primary joint replacement; or the first recorded procedure was a revision joint replacement as recorded in the Australian Orthopaedic Association (AOA) National Joint Replacement Registry (NJRR) – thus leaving 39,023 participants available for analysis.

### Anthropometric measurements

Height, weight, and waist and hip circumferences were measured once at baseline attendance for each participant according to written protocols based on standard procedures [22]. Weight was measured to the nearest 100 g using digital electronic scales. Height and waist and hip circumferences were measured to the nearest 1 mm using a stadiometer and a metal anthropometric tape, respectively. BMI was calculated as the weight in kilograms divided by the square of height in meters. The WHR was computed as the waist circumference divided by the hip circumference.

Bioelectrical impedance analysis was performed with a single frequency (50 kHz) electric current produced by a BIA-101A RJL system analyser (RJL systems, Detroit, MI, USA). Resistance and reactance were measured with subjects in a supine position. The nonadipose mass, hereafter termed fat-free mass, was estimated as 9.1536 + (0.4273 × height^2^/resistance) + (0.1926 × weight) + (0.0667 × reactance) for males, and as 7.7435 + (0.4542 × height^2^/resistance) + (0.119 × weight) + (0.0455 × reactance) for females [23]. The adipose mass, hereafter termed fat mass (FM = weight - fat-free mass), and the percentage fat (FM divided by weight) were subsequently calculated.

### Questionnaire measures

At baseline interview, questions were asked on date of birth, country of birth, smoking, alcohol consumption, current physical activity during leisure time, and highest level of education. At the second follow-up, the participants were asked questions enquiring about their first joint replacement surgery: Have you ever had a hip replacement? When did you have your first hip replacement? Have you ever had a knee replacement? When did you have your first knee replacement?

### Identification of incident primary knee and hip joint replacement

All participants gave written consent allowing access to their medical records. Cases were identified from the AOA NJRR.

The implementation of the AOA NJRR commenced in 1999 and was introduced in a staged state-by-state approach that was completed nationally by mid 2002. Victorian data collection commenced in 2001. The Registry monitors the performance and outcome of both hip and knee replacement surgery in Australia. It contains detailed information on the prostheses and surgical technique used and on the clinical situation that was used in for both primary and revision joint replacement [24]. By using detailed matching technology it is able to determine the success or otherwise of the joint replacement surgery. Although data collection for the NJRR is voluntary, the Registry receives cooperation from all hospitals undertaking joint replacement surgery [24].

The AOA NJRR validates its data using both internal systems and external data sources. The most important external data source is state health department data. Validation of registry data against health department recorded data involves a sequential multilevel matching process. Following the validation process and the retrieval of unreported records, the Registry collects the most complete set of data relating to hip and knee replacement in Australia [2].

Identifying information of MCCS participants – including first name, last name, date of birth, and gender – was provided to the staff at the AOA NJRR in order to identify those MCCS participants who had undergone a primary or revision joint replacement between 1 January 2001 which is when the Registry commenced Victorian data collection, and 31 December 2005. The matching was performed on these data provided using US Bureau of the Census Record Linkage software. Exact matches were identified and probabilistic matches were reviewed. The staff from the AOA NJRR forwarded this information to the MCCS and it was then added to the MCCS database.

The study was approved by The Cancer Council Victoria's Human Research Ethics Committee and the Standing Committee on Ethics in Research Involving Humans of Monash University.

### Statistical analysis

Cox proportional hazards regression models were used to estimate the hazard ratios (HRs) for first recorded primary joint replacement associated with each adiposity measure after adjustment for confounding variables. Follow-up for primary joint replacement (that is, calculation of person-time) began on 1 January 2001, and ended at date of first primary joint replacement for OA or date of censoring. Subjects were censored at either the date of first primary joint replacement performed for indications other than OA, the date of death, the date left Australia, or 31 December 2005 (the date that ascertainment of joint replacement by NJRR was complete), whichever came first.

Initially, all adiposity measures were categorized into approximate quartiles according to their sex-specific baseline distribution in the study population, and the association of each measure with risk of joint replacement was analysed separately, with the lowest quartile used as the referent category. The BMI was also categorized according to the widely used World Health Organization categories as follows: ≤ 24.9 kg/m^2^, 25.0 to 29.9 kg/m^2^, and ≥ 30.0 kg/m^2^. The waist circumference was further categorized as follows: for men, ≤ 93.9 cm, 94.0 to 101.9 cm, and ≥ 102.0 cm; and for women, ≤ 79.9 cm, 80.0 to 87.9 cm, and ≥ 88.0 cm [25]. For both BMI and waist circumference, the first predefined sex-specific category was the referent group. In addition, adiposity measures were fitted as continuous covariates to estimate linear trends on the log HR stratifying by gender. To estimate HRs separately for knee and hip replacement risk and to test for heterogeneity, Cox models based on competing risks were fitted using a data duplication method [26].

The following variables were considered as potential confounders: age, gender, country of birth (Australia, United Kingdom, Italy, Greece), and highest level of education (primary school, some high or technical school, completed high or technical school, and completed tertiary degree or diploma), since they have been shown to be related to the risk of joint replacement [27-29]. Other potential confounding variables (alcohol consumption (g/day), smoking (never, past, current), and physical activity at leisure (none, low, moderate, high)) were included in all of the definitive analyses if they changed the HRs of any of the adiposity measures by at least 5%. For each adiposity measure, all potential confounders were included in the model. To test whether associations between adiposity measures and joint replacement risk were modified by gender or BMI, interactions between these latter two variables and the adiposity measures were fitted, and tested using the likelihood ratio test.

Tests based on Schoenfeld residuals and graphical methods using Kaplan–Meier curves showed no evidence that proportional hazard assumptions were violated for any of the adiposity measures. *P *< 0.05 (two-sided) was considered statistically significant. All statistical analyses were performed using Stata (Intercooled Stata 9.2 for Windows; StataCorp LP, College Station, TX, USA).

## Results

A total of 1,009 primary joint replacements (541 knee replacements and 468 hip replacements) performed for OA were identified between 1 January 2001 and 31 December 2005. Descriptive statistics for selected characteristics of the study participants are presented in Table 1. The means of age and of all anthropometric measurements except for height were greater in those with a primary joint replacement compared with those with no joint replacement. Participants with a joint replacement were less likely to be born in Italy or Greece when compared with those with no joint replacement.

**Table 1:** Characteristics of the study population

	Primary knee replacement (n = 541)	Primary hip replacement (n = 468)	No primary joint replacement (n = 38,014)
Age of entering the MCCS (years)	60.3 ± 6.8	59.7 ± 7.1	54.8 ± 8.6
Age of entering the JR cohort (years)	68.1 ± 6.9	67.3 ± 7.2	62.6 ± 8.8
Age on JR (years)	70.7 ± 6.9	70.1 ± 7.2	
Women, number (%)	344 (63.6)	283 (60.5)	22,699 (59.7)
Height (cm)	164.7 ± 9.9	165.7 ± 8.8	165.0 ± 9.3
Weight (kg)	80.7 ± 14.9	75.5 ± 13.2	73.1 ± 13.6
Body mass index (kg/m^2^)	29.8 ± 5.1	27.5 ± 4.3	26.8 ± 4.4
Waist circumference (cm)	91.7 ± 12.8	86.3 ± 12.7	85.1 ± 12.8
Waist-to-hip ratio	0.85 ± 0.09	0.84 ± 0.10	0.84 ± 0.09
Fat mass (kg)	32.3 ± 10.4	27.8 ± 9.1	26.0 ± 9.1
Percentage fat (%)	39.7 ± 8.6	36.5 ± 8.3	35.3 ± 8.6
Country of birth, number (%)			
Australia	429 (79.3)	383 (81.8)	25,977 (68.3)
United Kingdom	41 (7.6)	32 (6.8)	2,774 (7.3)
Italy	45 (8.3)	39 (8.3)	4,999 (13.2)
Greece	26 (4.8)	14 (3.0)	4,264 (11.2)
Education level, number (%)			
Primary school	85 (15.8)	50 (10.7)	7,107 (18.8)
Some high school	255 (47.3)	192 (41.2)	14,422 (38.2)
Completed high school	112 (20.8)	102 (21.9)	7,814 (20.7)
Degree/diploma	87 (16.1)	122 (26.2)	8,426 (22.3)

Data reported as the mean ± standard deviation, except where indicated. MCCS, Melbourne Collaborative Cohort Study; JR, joint replacement.

Relationships between individual adiposity measures and the risk of primary joint replacement for OA, adjusted for age, gender, country of birth, and highest level of education, are presented in Table 2. Weight, BMI, waist circumference, WHR, FM, and percentage fat were all associated with an increased risk of primary joint replacement. When participants in the highest quartile of the adiposity measures were compared with those in the lowest quartile, the HRs were as follows: weight, 3.44 (95% confidence interval (CI) = 2.83 to 4.18); BMI, 3.44 (95% CI = 2.80 to 4.22); waist circumference, 2.77 (95% CI = 2.26 to 3.39); WHR, 1.46 (95% CI = 1.21 to 1.76); FM, 3.51 (95% CI = 2.87 to 4.30); and percentage fat, 2.99 (95% CI = 2.46 to 3.63). When using the predefined groupings of BMI and waist circumference and comparing the second and third categories with the first category, the HRs were 1.91 (95% CI = 1.62 to 2.24) and 3.08 (95% CI = 2.58 to 3.68) for BMI, and were 1.53 (95% CI = 1.31 to 1.79) and 2.17 (95% CI = 1.86 to 2.52) for waist circumference. Using the continuous form of the adiposity measures revealed similar inferences about risk of primary joint replacement as the categorical measures.

**Table 2:** Relationship between adiposity measures and risk of primary joint replacement

Variable	Number of cases in each quartile	Hazard ratio	95% confidence interval	*P *value
Weight (kg)				
Q1 (F <59.7, M <73.0)	136 (9,756)	1.00		
Q2 (F 59.7 to 66.2, M 73.0 to 79.7)	202 (9,485)	1.58	1.27 to 1.97	
Q3 (F 66.2 to 74.6, M 79.7 to 87.4)	269 (9,418)	2.17	1.77 to 2.67	
Q4 (F >74.6, M >87.4)	401 (9,329)	3.44	2.83 to 4.18	
Linear model (per 10 kg)		1.42	1.36 to 1.48	<0.001
Body mass index (kg/m^2^)				
Q1 (F <23.2, M <24.7)	128 (9,663)	1.00		
Q2 (F 23.2 to 25.9, M 24.7 to 26.8)	211 (9,511)	1.65	1.32 to 2.05	
Q3 (F 25.9 to 29.3, M 26.8 to 29.2)	302 (9,458)	2.48	2.01 to 3.05	
Q4 (F >29.3, M >29.2)	367 (9,354)	3.44	2.80 to 4.22	
Linear model (per 5 kg/m^2^)		1.60	1.51 to 1.70	<0.001
Waist circumference (cm)				
Q1 (F <71.0, M <87.0)	136 (9,943)	1.00		
Q2 (F 71.0 to 78.0, M 87.0 to 93.0)	222 (9,612)	1.59	1.29 to 1.97	
Q3 (F 78.0 to 87.0, M 93.0 to 99.0)	290 (9,399)	2.07	1.68 to 2.54	
Q4 (F >87.0, M >99.0)	360 (9,025)	2.77	2.26 to 3.39	
Linear model (per 10 cm)		1.38	1.31 to 1.45	<0.001
Waist-to-hip ratio				
Q1 (F < 0.74, M < 0.88)	193 (9,553)	1.00		
Q2 (F 0.74 to 0.78, M 0.88 to 0.92)	234 (9,516)	1.13	0.94 to 1.37	
Q3 (F 0.78 to 0.82, M 0.92 to 0.96)	264 (9,498)	1.23	1.02 to 1.48	
Q4 (F > 0.82, M > 0.96)	317 (9,410)	1.46	1.21 to 1.76	
Linear model (per 0.1 unit)		1.23	1.12 to 1.35	<0.001
Fat mass (kg)				
Q1 (F <21.1, M <18.4)	125 (9,586)	1.00		
Q2 (F 21.1 to 26.5, M 18.4 to 23.0)	209 (9,502)	1.71	1.37 to 2.13	
Q3 (F 26.5 to 32.9, M 23.0 to 27.9)	273 (9,438)	2.26	1.83 to 2.80	
Q4 (F >32.9, M >27.9)	398 (9,312)	3.51	2.87 to 4.30	
Linear model (per 10 kg)		1.61	1.52 to 1.71	<0.001
Percentage fat (%)				
Q1 (F <35.3, M <25.0)	143 (9,568)	1.00		
Q2 (F 35.3 to 40.1, M 25.0 to 28.9)	205 (9,506)	1.45	1.17 to 1.80	
Q3 (F 40.1 to 44.6, M 28.9 to 32.6)	260 (9,451)	1.86	1.51 to 2.28	
Q4 (F >44.6, M >32.6)	397 (9,313)	2.99	2.46 to 3.63	
Linear model (per 10%)		2.01	1.81 to 2.22	<0.001

All models using sex-specific quartiles of adiposity measures adjusted for age, gender, country of birth, and highest level of education. All models using continuous adiposity measures adjusted for age, country of birth, and highest level of education, stratifying by gender, representing an approximate change of one standard deviation for each of the adiposity measures. Q1, first quartile; Q2, second quartile; Q3, third quartile; Q4, fourth quartile; F, female; M, male.

No evidence was found for a departure from a linear association between adiposity measures and joint replacement risk within our sex-specific observed range of adiposity measures (see Table 2 for sex-specific quartile cutoff points). There was no threshold effect within the sex-specific observed range of adiposity measures.

The specificities at each quartile cutoff point were similar for all of the adiposity measures. The sensitivities at each quartile cutoff point, however, were lower for WHR than for the other adiposity measures (data not shown).

The associations between adipose mass (FM and percentage fat) and central adiposity (waist circumference and WHR) measures and joint replacement risk were not modified by BMI. Gender did not modify the associations between adiposity measures and the risk of joint replacement, and similar results were observed when men and women were examined separately (Table 3).

**Table 3:** Relationship between adiposity measures and risk of primary joint replacement by gender

	Females		Males	
		
Variable	Hazard ratio (95% confidence interval)	*P *value	Hazard ratio (95% confidence interval)	*P *value
Weight, kg				
Q1 (F <59.7, M <73.0)	1.00		1.00	
Q2 (F 59.7 to 66.2, M 73.0 to 79.7)	1.84 (1.37 to 2.46)		1.30 (0.93 to 1.80)	
Q3 (F 66.2 to 74.6, M 79.7 to 87.4)	2.69 (2.04 to 3.54)		1.58 (1.15 to 2.16)	
Q4 (F >74.6, M >87.4)	4.12 (3.16 to 5.37)		2.60 (1.93, 3.49)	
Linear model (per 10 kg)	1.42 (1.35 to 1.50)	<0.001	1.41 (1.31 to 1.52)	<0.001
Body mass index, kg/m^2^				
Q1 (F <23.2, M <24.7)	1.00		1.00	
Q2 (F 23.2 to 25.9, M 24.7 to 26.8)	1.85 (1.38 to 2.47)		1.40 (1.00 to 1.97)	
Q3 (F 25.9 to 29.3, M 26.8 to 29.2)	2.68 (2.03 to 3.54)		2.23 (1.63 to 3.06)	
Q4 (F >29.3, M >29.2)	3.87 (2.94 to 5.09)		2.85 (2.08 to 3.90)	
Linear model (per 5 kg/m^2^)	1.55 (1.45 to 1.66)	<0.001	1.76 (1.56 to 1.98)	<0.001
Waist circumference, cm				
Q1 (F <71.0, M <87.0)	1.00		1.00	
Q2 (F 71.0 to 78.0, M 87.0 to 93.0)	1.72 (1.29 to 2.28)		1.45 (1.05 to 2.02)	
Q3 (F 78.0 to 87.0, M 93.0 to 99.0)	2.24 (1.70 to, 2.94)		1.84 (1.33 to 2.53)	
Q4 (F >87.0, M >99.0)	3.03 (2.31 to 3.97)		2.39 (1.76 to 3.24)	
Linear model (per 10 cm)	1.36 (1.27 to 1.44)	<0.001	1.41 (1.28 to 1.55)	<0.001
Waist-to-hip ratio				
Q1 (F <0.74, M <0.88)	1.00		1.00	
Q2 (F 0.74 to 0.78, M 0.88 to 0.92)	1.25 (0.98 to 1.59)		0.94 (0.69 to 1.29)	
Q3 (F 0.78 to 0.82, M 0.92 to 0.96)	1.25 (0.98 to 1.59)		1.16 (0.86 to 1.57)	
Q4 (F > 0.82, M > 0.96)	1.32 (1.04 to 1.68)		1.62 (1.22 to 2.15)	
Linear model (per 0.1 unit)	1.16 (1.03 to 1.30)	0.01	1.36 (1.16 to 1.60)	<0.001
Fat mass, kg				
Q1 (F <21.1, M <18.4)	1.00		1.00	
Q2 (F 21.1 to 26.5, M 18.4 to 23.0)	1.85 (1.38 to 2.49)		1.53 (1.09 to 2.15)	
Q3 (F 26.5 to 32.9, M 23.0 to 27.9)	2.60 (1.96 to, 3.45)		1.84 (1.33 to 2.55)	
Q4 (F >32.9, M >27.9)	4.04 (3.09 to 5.29)		2.80 (2.06 to 3.81)	
Linear model (per 10 kg)	1.58 (1.47 to 1.69)	<0.001	1.69 (1.51 to 1.89)	<0.001
Percentage fat, %				
Q1 (F <35.3, M <25.0)	1.00		1.00	
Q2 (F 35.3 to 40.1, M 25.0 to 28.9)	1.47 (1.11 to 1.96)		1.43 (1.04 to 1.99)	
Q3 (F 40.1 to 44.6, M 28.9 to 32.6)	2.12 (1.62 to 2.77)		1.51 (1.09 to 2.08)	
Q4 (F >44.6, M >32.6)	3.29 (2.55 to 4.24)		2.56 (1.90 to 3.45)	
Linear model (per 10%)	1.99 (1.76 to 2.26)	<0.001	2.00 (1.67 to 2.39)	<0.001

All models adjusted for age, country of birth, and highest level of education. Linear models represent an approximate change of one standard deviation for each of the adiposity measures. Q1, first quartile; Q2, second quartile; Q3, third quartile; Q4, fourth quartile; F, female; M, male.

When primary knee and hip replacement were examined separately, all of the individual adiposity anthropometric measures were associated with an increased risk of both primary knee and hip replacement – except that WHR was not significantly associated with the risk of primary hip replacement (Table 4). Moreover, all of the adiposity measures were stronger risk factors for knee rather than hip replacement. For example, for every 5 unit increase in BMI, the HRs were 1.88 (95% CI = 1.76 to 2.00) for knee replacement and 1.26 (95% CI = 1.15 to 1.38) for hip replacement (*P *< 0.001 for heterogeneity of HRs) (Table 4).

**Table 4:** Relationship between adiposity measures and risk of primary knee and hip replacement

	Primary knee replacement (n = 541)		Primary hip replacement (n = 468)		Heterogeneity of hazard ratios
			
	Hazard ratio (95% confidence interval)	*P *value	Hazard ratio (95% confidence interval)	*P *value	(*P *value)
Weight (per 10 kg)	1.58 (1.51 to 1.65)	<0.001	1.22 (1.15 to 1.30)	<0.001	<0.0001
Body mass index (per 5 kg/m^2^)	1.88 (1.76 to 2.00)	<0.001	1.26 (1.15 to 1.38)	<0.001	<0.0001
Waist circumference (per 10 cm)	1.62 (1.53 to 1.72)	<0.001	1.10 (1.01 to 1.19)	0.03	<0.0001
Waist-to-hip ratio (per 0.1 unit)	1.43 (1.29 to 1.58)	<0.001	1.01 (0.85 to 1.19)	0.92	0.0004
Fat mass (per 10 kg)	1.88 (1.76 to 2.00)	<0.001	1.29 (1.18 to 1.41)	<0.001	<0.0001
Percentage fat (per 10%)	2.84 (2.47 to 3.26)	<0.001	1.37 (1.19 to 1.57)	<0.001	<0.0001

All models adjusted for age, country of birth, and highest level of education, stratifying by gender, representing an approximate change of one standard deviation for each of the adiposity measures.

## Discussion

We have demonstrated a threefold to fourfold increased risk of primary hip and knee joint replacement for OA, when comparing the fourth quartile with the first quartile, for weight, BMI, FM, and percentage fat. The waist circumference and WHR were less strongly associated with the risk. When knee and hip replacements were examined separately, all adiposity measures persisted as risk factors for joint replacement at either anatomical site – with the exception of WHR, which was not significantly associated with hip replacement risk. Moreover, when comparing the strength of the associated risks between the adiposity measures and knee and hip joint replacement, all adiposity measures were stronger risk factors for knee replacement rather than hip joint replacement.

There are few previous studies examining the relationship between directly measured adipose mass or the distribution of adipose mass and the risk of joint replacement. Most studies have employed BMI as a measure of obesity, and have shown a consistently positive association between BMI and the risk of both knee replacements [13,14] and hip replacements [13-17] for OA – as has the present study. In addition to examining the association with BMI, it would be necessary to examine whether the pattern of fat distribution or body composition affects this risk [18], in order to explore the mechanism that may explain the association between obesity and the increased risk of joint replacement due to severe OA. In a cohort of the Swedish general population (the Malmo Diet and Cancer study), Lohmander and colleagues found that all body mass measures were significant risk factors for knee and hip OA leading to joint replacement [20]. Consistent with this study, we also showed that measures of adipose mass (FM and percentage fat) were associated with an increased risk of primary knee and hip joint replacement 10 to 15 years after their measurement. Whilst both measures of central adiposity (waist circumference and WHR) were associated with an increased risk of primary knee replacement, only waist circumference but not WHR was significantly associated with the risk of primary hip replacement. Moreover, for all measures of obesity, stronger evidence was observed for the knee than for the hip.

The Malmo Diet and Cancer study [20] and our study are the only two prospective studies of which we have knowledge that have investigated the association of different adiposity measures (including direct measurement of percentage fat using bioelectrical impedance) with joint replacement. The findings of the Malmo Diet and Cancer study (n = 27,960) have been confirmed in our larger study (n = 39,023). Our study also included about 24% of participants who were Southern European migrants, whereas the Malmo Diet and Cancer study excluded participants with a lack of Swedish language skills, thus resulting in a more homogeneous population. This significantly strengthens the findings since similar results have been found in different populations, suggesting that the association is more likely to be causal since it is unlikely that both studies were subject to the same type of errors (chance, bias or confounding).

The mechanism for the associations between adiposity measures and the risk of primary knee and hip joint replacement is unclear, but may be due to both biomechanical and metabolic factors. The adipose mass, by virtue of its added body mass, contributes to an increased joint loading, which may increase the risk of OA progression and subsequent joint replacement performed for severe end-stage OA. This biomechanical hypothesis may be most evident at the knee – given the anatomical disadvantage that the knee joint lacks a stable bony configuration compared with the hip, whereby load is disproportionately distributed to the medial tibiofemoral compartment during dynamic tasks – and it has been shown that much of the effect of BMI on the severity of medial tibiofemoral OA was explained by varus malalignment [30]. The association between fat distribution and the risk of knee and hip OA has been investigated in different study populations with inconsistent results. While some studies showed positive associations [31], other studies showed no association [5,9,32,33].

Nevertheless, metabolic factors are also likely to be important since we have shown that waist circumference and WHR, the surrogate measures of central adiposity and known risk factors for the metabolic syndrome [34], were more strongly associated with the risk of knee replacement than hip replacement. Indeed adipose tissue, which was once thought to be a passive store of energy, is now considered an endocrine organ, releasing a multitude of factors, including cytokines such as TNFα and IL-6, as well as adipokines, such as leptin, adiponectin and resistin [35]. Both TNFα and IL-6 have been implicated in cartilage destruction in OA [36,37], while leptin is a key regulator of chondrocyte metabolism and plays an important role in the pathophysiology of OA [38]. Such findings demonstrate the potential role of metabolic factors related to adiposity in the context of OA and, ultimately, of joint replacement.

The WHR is a surrogate measure of central adiposity that includes the visceral and abdominal subcutaneous depots. Recent data have shown some biological differences between intraabdominal visceral fat and peripheral subcutaneous fat [39]. Visceral adipose tissue and its adipose-tissue resident macrophages produce more proinflamatory cytokines, like TNFα and IL-6, and less adiponectin [39]. Leptin secretion is greater from subcutaneous than from visceral fat tissue [40]. A limitation of the WHR is that it is not able to discern between the metabolically and physically different types of fat. In addition, the WHR becomes even less reliable in people who have both greater central and gynoid fat, and therefore may lead to an underestimation of observed associations. This underestimation may in part explain the lower sensitivity and weaker association of WHR with joint replacement than the other adiposity measures.

We had virtually complete follow-up in this prospective study as the identification of incident primary knee and hip replacement was done by record linkage to the NJRR, which has complete coverage of the cohort participants. While the recruitment of MCCS participants and data collection commenced in 1990 to 1994, the NJRR started joint replacement data collection in Victoria in 2001. We therefore do not have complete and reliable joint replacement data for the study population prior to 2001. Although we excluded those MCCS participants who reported a joint replacement prior to 1 January 2001 at the second follow-up, this information may be unreliable and is only known for 68% of the original cohort. As a result, some misclassification of joint replacement status may have occurred – although it is likely to have been nondifferential in relation to the adiposity measures, which may have underestimated the strength of any observed associations. The MCCS did not collect data on occupational activities such as bending and lifting, and thus we were unable to adjust for these factors in the analysis. Although total joint replacement is used as a proxy for severe symptomatic OA, the utilization of joint replacement in the treatment of OA may be influenced by a number of factors such as access to healthcare, physician bias, and patient-level factors, in addition to disease severity [41]. We therefore adjusted for age, gender, country of birth, and highest level of education in the analysis to counter this issue.

A particular issue for bioelectric impedance analysis is the absence of a standard equation to estimate the fat-free mass. We chose a formula developed using subjects of similar ethnicity, age, and BMI distribution to the MCCS population [23] that was validated using sound statistical techniques. There is evidence that body hydration, a status difficult to assess in large epidemiological studies, has a strong effect on the estimation of FM based on bioelectric impedance analysis [42]. Any between-subject variability in hydration level in the current study would therefore have resulted in greater attenuation of the relationship between FM and the risk of primary joint replacement. Another concern is that the measurement error in the anthropometric variables would have underestimated the associations observed in the study, and this effect would be greatest for the bioelectric impedance analysis-based measures.

## Conclusion

In summary, the risk of primary knee replacement and hip joint replacement for OA appears to be related to BMI, both adipose mass and central adiposity, whereas the WHR was significant for knee replacement but not hip replacement. This suggests both biomechanical and metabolic mechanisms associated with adiposity contribute to the risk of joint replacement, with stronger evidence at the knee rather than at the hip. The obesity epidemic occurring in developed countries is likely to have a significant impact on the future demands for knee and hip replacements for OA, and understanding the mechanism of action will be important in effective prevention of OA.

## Abbreviations

AOA: Australian Orthopaedic Association; BMI: body mass index; CI: confidence interval; FM: fat mass; HR: hazard ratio; IL: interleukin; MCCS: Melbourne Collaborative Cohort Study; NJRR: National Joint Replacement Registry; OA: osteoarthritis; TNFα: tumour necrosis factor alpha; WHR: waist-to-hip ratio.

## Competing interests

The authors declare that they have no competing of interests.

## Authors' contributions

YW participated in the design of the study, performed the statistical analysis and the interpretation of data, and drafted the manuscript. JAS participated in the acquisition of data, helped to perform the statistical analysis, and reviewed the manuscript. AEW and AJT helped the interpretation of data, and reviewed the manuscript. DRE, GGG, and SG participated in the design of the study and the acquisition of data, and reviewed the manuscript. FMC participated in the design of the study, helped with the interpretation of data, and reviewed the manuscript. All authors read and approved the final manuscript.
